# Viral genome sequencing methods: benefits and pitfalls of current approaches

**DOI:** 10.1042/BST20231322

**Published:** 2024-05-15

**Authors:** Natasha Jansz, Geoffrey J. Faulkner

**Affiliations:** 1 Mater Research Institute - University of Queensland, TRI Building, Woolloongabba, QLD 4102, Australia; 2 Queensland Brain Institute, University of Queensland, Brisbane, QLD 4072, Australia

**Keywords:** DNA sequencing, genomics, virology

## Abstract

Whole genome sequencing of viruses provides high-resolution molecular insights, enhancing our understanding of viral genome function and phylogeny. Beyond fundamental research, viral sequencing is increasingly vital for pathogen surveillance, epidemiology, and clinical applications. As sequencing methods rapidly evolve, the diversity of viral genomics applications and catalogued genomes continues to expand. Advances in long-read, single molecule, real-time sequencing methodologies present opportunities to sequence contiguous, haplotype resolved viral genomes in a range of research and applied settings. Here we present an overview of nucleic acid sequencing methods and their applications in studying viral genomes. We emphasise the advantages of different viral sequencing approaches, with a particular focus on the benefits of third-generation sequencing technologies in elucidating viral evolution, transmission networks, and pathogenesis

## Introduction

A virus is a submicroscopic, obligate intracellular parasite that comprises a DNA or RNA genome. In the extracellular phase, the viral genome is enclosed within a protective structure called a virion, which enables its delivery to its target cell. In the intracellular phase of the viral life cycle, the viral genome hijacks the host's cellular machinery to direct its replication, packaging and propagation. The viral genome is the predominant site of intracellular host-viral interactions. Understanding the viral genome provides valuable insights into the viral life cycle, its evolutionary history, and interactions between the virus and its host.

As nucleic acid sequencing methodologies have emerged, viral genomes have been among the primary targets for adoption of these new technologies. The first protein coding sequence resolved was that of Bacteriophage MS2, by RNAse digestion and chromatography [1]. In 1977, the first DNA genome sequenced using the ‘plus-minus method’ was the 5368 bp genome of Bacteriophage φX174 [2,3]. Around the same time, so-called first generation sequencing methodologies were developed, and within a few years, Frederick Sanger and his team sequenced and assembled the significantly larger 48 502 bp Bacteriophage λ genome [4]. Due to their relatively small genome size, sequencing of viral isolates became a routine practice in the 1980s [5,6]. Fast forward to the 21st century, and the emergence of high throughput and single molecule sequencing methodologies has seen millions of viral genomes resolved and catalogued, ranging from cultured isolates in laboratory settings to uncultivated viruses sequenced via unbiased metagenomics approaches.

Here we will describe the principles underlying common genome sequencing methodologies and their utilisation in virology, with a particular focus on long-read sequencing technologies. We will draw examples from molecular virology, metagenomics, genomic epidemiology and surveillance, and clinical applications. We will also describe limitations of common sequencing approaches, and elegant strategies to overcome such pitfalls.

## Sequencing methodologies

**Figure 1.**
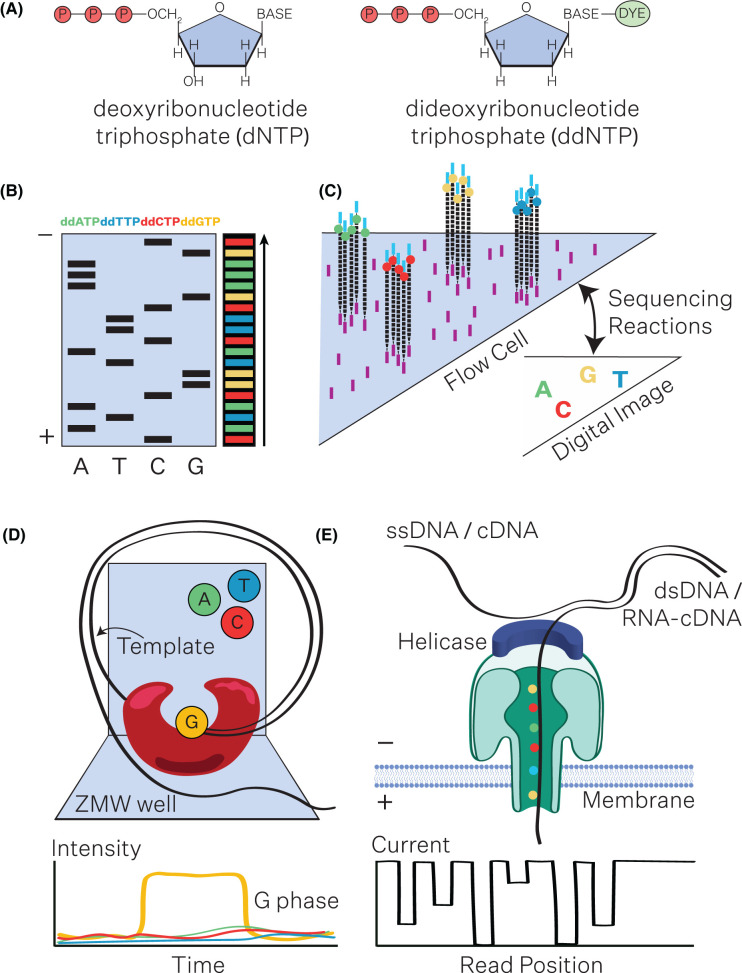
Principles underlying common sequencing methods. (**A**) Chain termination sequencing makes use of dideoxynucleotides (ddNTPs). ddNTPs are similar in structure to deoxynucleotides (dNTPs), but lack the 3′ hydroxyl group. The ddNTPs may be radioactively or fluorescently labelled. When a ddNTP is incorporated into a DNA strand, DNA synthesis stops. In a sequencing reaction, dNTPs are present in excess and chain elongation proceeds normally until DNA polymerase adds a labelled ddNTP, arresting elongation. (**B**) Following the sequencing reaction, the products of varying lengths are separated by either gel (left) or capillary electrophoresis (right), and can be visualised by autoradiography or fluorescence to infer the DNA sequence. (**C**) Illumina dye sequencing is a second generation sequencing-by-synthesis approach that involves fragmenting DNA inputs, and ligating sequencing adapters to the ends of fragments. The fragments can then hybridise to a solid flow cell by the adapter sequences, where they are amplified into a clonal cluster, which serves as a sequencing template. The sequencing reaction includes fluorescently labelled dNTPs. As each base is incorporated into the newly synthesised strand, the flow cell is imaged, and the specific emission of each cluster recorded to identify the newly incorporated base. The fluorescently labelled nucleotide serves as a ‘reversible terminator’, as the label can be enzymatically cleaved after each sequencing reaction, enabling the next round of dNTP incorporation. (**D**) SMRT HiFi sequencing is a single-molecule, long-read sequencing technology. Circularised fragments of DNA are prepared and washed over a nanofluidic chip containing millions of wells called zero-mode waveguides (ZMWs). A single molecule of circularised DNA is associated with a DNA polymerase (red) and immobilised at the bottom of a ZMW. From inside the ZMW, labelled nucleotides are incorporated into a newly synthesised strand. SMRT-seq uses nucleotides containing a fluorescent label on the phosphate chain of the nucleotide rather than on the base. Incorporated nucleotides are detected in real time, based on the associated fluorophore released upon cleavage of the phosphate chain, to infer the DNA sequence in each ZMW. (**E**) Nanopore sequencing is a direct real-time, single-molecule, long-read sequencing method. Nanopore flow cells contain an array of transmembrane nanopores (green) embedded in an electro-resistant membrane (blue). Each nanopore connects to an electrode, which measures the electric current that flows through the nanopore. When a nucleic acid molecule is guided through a nanopore by a helicase (navy blue), the current is disrupted resulting in a characteristic ‘squiggle’. The nucleic acid sequence can then be inferred from the squiggle in real time, using basecalling algorithms based on neural networks.

Genome sequencing is broadly demarcated into three generations, each representing a substantive shift in sequencing capabilities. While specificities and applications vary, most sequencing platforms are based on a common underlying chemistry (Figure 1). Here we will outline the principles underlying the most widely adopted sequencing platforms today: sequencing-by-synthesis; and direct nanopore sequencing (Table 1).

**Table 1.** Comparison between common first, second and third generation sequencing methodologies

Platform	Accuracy	Maximum read length	Input (gDNA)	Output (Gb)	Multiplexing	Direct detection of modified bases	Benefits
Chain termination	99.99%	1000 bp	1–3 µg (phage DNA)	0.00009	No	No	Cost of library preparation makes it a suitable choice for targeted sequencing in low resource environments, can pair with chemical conversion protocols to indirectly detect modified bases of clones
Illumina NextSeq 2000	99.9%	2 × 300 bp	10 ng	≤540	Yes	No	Enables multiplexing of up to 384 samples, most functional genomics assays designed for this platform, a suite of enrichment or conversion protocols to infer nucleotide and chromatin modifications
Illumina (NovaSeq 6000)	99.9%	2 × 250 bp	10 ng	≤3000	Yes	No	Cheapest cost-per-base sequencing method, Enables multiplexing of up to 384 samples, most functional genomics assays designed for this platform
Illumina (Complete Long Reads)	99.9%	10 kb	10 ng	≤3000	Yes	No	Viable long-read sequencing option for low-input samples, can be paired with enrichment protocols
SMRT-seq	99.87% HiFi reads	100 kb	2 µg	≤90	Yes	Yes, although low signal-to-noise	Most accurate, long-read, single molecule, real-time method, industry standard for long-read transcriptome sequencing, standaradised analysis pipelines
Nanopore (MinION)	<99.5% simplex reads	4 Mb	1 µg	10–20 (48 theoretical max.)	Yes	Yes	Portability, minimal initial investment, PCR-free library preparations, direct RNA sequencing, real-time analysis, single molecule sequencing, duplex reads can increase accuracy at the cost of depth
Nanopore (PromethION)	<99.5% simplex reads	4 Mb	1 µg	100–200 (277 theoretical max.)	Yes	Yes	PCR-free library preparations, direct RNA sequencing, real-time analysis, single molecule sequencing, duplex reads can increase accuracy at the cost of depth

Sequencing-by-synthesis approaches, based on the chain termination sequencing method, rely on the sequential detection of labelled nucleotides incorporated into a primed single-stranded template by a polymerase [7]. In essence, chain termination makes use of dNTP analogues, ddNTPs, that halt the extension of the newly synthesised DNA strand upon incorporation (Figure 1A). Polymerase reactions are carried out on a clonal DNA sample, with a mix of normal triphosphates in excess of terminating triphosphates, and radiolabeled triphosphates. The resulting mixture can be resolved on a denaturing gel, allowing inference of the DNA sequence (Figure 1B). This approach enabled sequencing of fragments up to 1000 bp in length, limited by the ability to resolve large fragments of DNA that differ by just a nucleotide in length. Nonetheless, shotgun sequencing approaches, in which short fragments sequenced by the chain termination method are assembled by overlapping sequences, enabled the assembly of megabase scale genomes, first providing reference assemblies for commonly used model organisms [8,9]. Modifications of the chain termination method, including fluorescent labelling, capillary electrophoresis and automation enabled large scale uptake of the method as a first generation sequencing technology, which is still widely used today [10–13].

Second (or next) generation sequencing (NGS) platforms were marked by the massive parallelisation of sequencing reactions, allowing high-throughput sequencing of thousands of fragments simultaneously [14,15]. A key departure from first generation sequencing is the ability to sequence a mixed population of DNA on NGS platforms. NGS technologies are largely based on sequencing-by-synthesis chemistries, in which DNA fragments are immobilised in clusters on a surface, and incorporation of labelled nucleotides are read out in real time [16–18]. Various NGS approaches have been summarised elsewhere [19], but the most widely adopted high throughput sequencing method has been Illumina dye sequencing (Figure 1C). The increased throughput and reduced cost per base afforded by NGS has enabled not just genome sequencing, but also profiling of the transcriptome, epigenome, and exome of samples, through indirect enrichment or conversion protocols [20,21]. While considerably more high-accuracy sequencing data can be generated in a typical NGS sequencing run, NGS yields shorter read lengths, typically 75–600 bp, due to a length dependent phenomenon known as dephasing, when polymerase errors accumulate in each sequencing cluster [15]. The sequencing of shorter fragments has meant that NGS techniques are typically suited to analysis against previously assembled reference genomes rather than *de novo* assembly.

Third generation sequencing technologies, by contrast, are much better suited to do novo genome sequencing. Third generation platforms offer long-read, single molecule, real time sequencing, enabled by two quite different underlying chemistries. Single-molecule real-time (SMRT) sequencing operates on the principle of sequencing-by-synthesis, however the polymerase rather than nucleic acid is immobilised in the bottom of a confinement structure, which enables real-time observation of the incorporation of a single fluorescently labelled nucleotide (Figure 1D) [22–24]. SMRT-seq can generate high-fidelity (HiFi) reads through circular consensus sequencing, to achieve base-level resolution with 99.9% single-molecule accuracy. Nanopore sequencing represents a departure from traditional sequencing-by-synthesis approaches, enabling direct sequencing of nucleic acid molecules. Nanopore sequencing relies on electrophoresis to pass a nucleic acid molecule through a nanopore, which is embedded in a biological or solid state membrane in an electrolyte solution [25]. As the single molecule moves through the pore, each base elicits a specific change in the ionic current moving through the pore, allowing inference of the sequence from the modulation of the current in real-time (Figure 1E) [26]. Both approaches have seen an increase in read length, with SMRT-seq producing an average read length of 30 kb (up to 100 kb), and nanopore read lengths are theoretically limited by the size of the molecule itself, with reports of a 4.2 Mb long read [27,28]. Additionally, nanopore sequencing allows for direct detection of modified DNA bases, eliminating the need for artefact-prone physical enrichment or conversion protocols [29]. The kinetics of base incorporation can also be used to predict DNA modifications by SMRT-seq; however the signal-to-noise ratio for 5mC inference is low, and relatively high coverage for calling modifications is required [30,31]. Nanopore sequencing can directly sequence RNA molecules, removing the need to generate cDNA libraries for sequencing [32]. Third generation sequencing platforms currently offer reduced throughput and raw read accuracy. However, regular improvements in the underlying biochemistry and computational models for analysis, mean that accuracy is continuously improving, and fast approaching NGS levels [28]. Illumina has adapted NGS protocols to mutate and amplify long molecules of DNA prior to fragmentation and sequencing on existing sequencing-by-synthesis platforms, enabling computational reconstruction of single-molecule, long-reads. This synthetic approach generates sequences with an average read length of 5–7 kb, and cannot be analysed in real time, nor resolve modified bases. However, 100-fold lower input requirements, and increased accuracy and throughput, makes Illumina long-read sequencing a viable option for long-read single-molecule sequencing. At the time of writing this review, Pacific Biosciences has proprietary rights to SMRT-sequencing technologies, and while there are many companies that have developed commercial nanopore sequencing platforms, the most widely used has been developed by Oxford Nanopore Technologies (ONT).

## Utilities of sequencing in virology

### Genome sequencing and assembly

**Figure 2.**
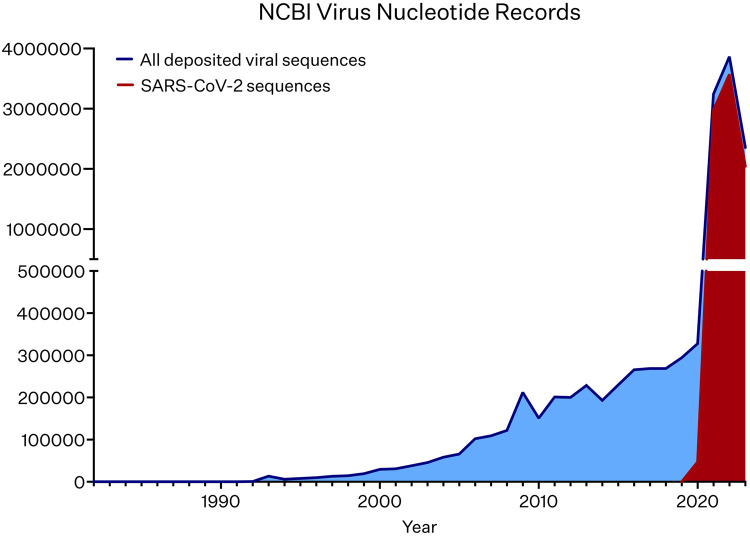
The number of NCBI Virus nucleotide records (*y*-axis) released over time (*x*-axis) from 1982 to 2023 [6]. Total deposited viral sequences are plotted in blue, and all SARS-CoV-2 nucleotide sequences deposited on NCBI from 2020 to 2023 are plotted in red.

A fundamental application of sequencing in virology is whole genome sequencing and assembly. Assembled viral genome sequences have steadily accumulated over the decades, with a notable surge since 2020 (Figure 2) [5,6,33], due to the rapid accumulation of the SARS-CoV-2 genomes in response to the global pandemic of 2020 [34]. Initially, cultured viral genomes were primary targets for characterisation using first-generation sequencing methods, benefiting from their small size and genetic uniformity among isolates. In fact, until 1995 the only completely resolved genomes belonged to viruses and organelles [5]. Most large scale viral sequencing projects have been initiated based on prior knowledge of an existing sequence, followed by genome walking strategies [35]. While second generation sequencing approaches allow for unbiased genome sequencing, short read lengths require analysis against existing genome assemblies, or assembly by chain termination sequencing in parallel. For this reason, there was not a rapid initial uptake in NGS technologies by virologists. Single-molecule long-read sequencing platforms offer unparalleled advantages when applied to viral genome assembly. Whereas *de novo* genome sequencing by NGS and chain-termination methods necessitated assembly from contigs, short fragments of DNA containing overlapping sequences, long-read platforms are frequently able to produce contiguous, end-to-end sequences spanning entire viral genomes, often bypassing the need for assembly altogether. This is particularly adventitious when it comes to assembling low complexity regions of the genome, as well as structural and copy-number variation, which is hard to resolve from clonal or population level short-read assemblies. Single-molecule sequencing also allows for individual genomes to be resolved within a mixed population, which not only elucidates variation and selection within a population, but can also reveal important functional implications. For example, recurrent integration of oncogenic human papillomavirus (HPV) genomes into host chromosomes is a feature of HPV-associated cancers [36]. Integrants can often be found as tandem repeats of the viral genome, which has been shown to have a functional impact on both host oncogene and viral expression [36]. Assembly methods from long-read sequencing have been able to better resolve recurrent insertions of HPV *in vitro* and in patient samples, to clarify structural complexities, including copy number duplications [37–39].

Another consideration in the application of sequencing technologies in virology is the diverse nature of viral genomes. Viruses can possess DNA or RNA genomes, including retroviruses that undergo an intracellular DNA intermediary phase. These genomes may be single-stranded or double-stranded, positive or negative-sense, segmented or contiguous, and linear or circular. The diversity in viral genomes necessitates different approaches to library preparation and sequence interpretation. For example, segmented genomes such as Influenza were once separated and purified by mass prior to sequencing and assembly, or paired with RACE data by NGS approaches to map the genome termini [40–42]. Similarly, various rolling circle amplification approaches have been employed to enrich and sequence circular ssDNA genomes, such as those of Geminiviridae [43,44]. While long-read sequencing technologies offer a more uniform approach to sequencing most viral genomes, they are particularly beneficial for viral RNA genomes [45]. Sequencing-by-synthesis approaches require a DNA template for sequencing, which for RNA genomes, necessitates a cDNA conversion step [46–48]. Unlike DNA genomes, which are relatively stable, RNA genomes are incredibly plastic. High mutation and recombination rates associated with RNA-dependent RNA polymerase creates a mixed populations of closely related viral quasispecies [49]. Such high variability even within a ‘clonal’ population, has proved challenging for the design of pan-reactive primers to reliably generate and amplify viral cDNAs. Furthermore, short-read sequencing technologies are ill-suited to reconstruct many diverse full-length haplotypes of RNA viral genomes [50].

Nanopore direct RNA sequencing (DRS) mirrors many aspects of nanopore DNA sequencing; a helicase attached to an RNA-DNA molecule guides the native RNA through an RNA specific nanopore upon voltage application [32]. As with DNA sequencing, DRS provides single molecule resolution of RNA sequences and the ability to detect nucleotide modifications. In principle, use of DRS to study viral gnomes should improve on NGS methodologies, as they bypass the need for a cDNA sequencing template, prior knowledge of the genome for amplification, and genome assembly from highly fragmented sequences [51–53]. Furthermore, the sequencing of contiguous genomes enables phasing of polymorphisms to resolve haplotypes within a population [52]. However, practical limitations have hindered the widespread adoption of nanopore DRS. Namely, limitations in basecall and RNA modification accuracy, high input RNA quality and quantity requirements, and the inability to read through full-length transcripts [54,55]. At the end of 2023, updated RNA nanopore and library preparation chemistries were made available by ONT, which promise to deliver increased yield, accuracy and length. At the time of writing, publicly available data to assess such improvements is scarce, however emerging studies adopting updated DRS chemistries are promising, delivering results that are on par with ONT cDNA sequencing [56]. As was the case with single molecule DNA sequencing, DRS accuracy will undoubtedly improve as the technology matures. For now, the industry-standard for long-read RNA sequencing remains cDNA library preparation and sequencing by nanopore or SMRT-seq. While cDNA conversion does not conserve RNA modifications and is prone to artefact generation by error prone reverse transcriptase, the increased accuracy and throughput afforded by cDNA sequencing better serves most metagenomics and viral assembly projects [57]. Both approaches typically employ a poly-A strategy for cDNA generation and amplification, and as such exhibit a 3′ bias [54]. This bias is more pronounced with nanopore cDNA sequencing, owing not only to limitations imposed by reverse transcription, but also properties of the pores themselves, such as inaccuracies at the terminal ends of reads, and incomplete translocation through the pores to truncate reads [58]. While computational methods are emerging to filter truncated reads, they often rely on prior knowledge of genome features [59,60]. Although long-read cDNA sequencing approaches are not yet capable of producing full-length viral genomes, this is a keen area of development for both PacBio and ONT. Of note is the recent implementation of the Kinnex approach by PacBio, which creates large cDNA concatemer arrays capable of generating full-length PacBio HiFi sequences of RNA molecules [61]. A similar array approach has been reported by ONT users, and although not widely implemented, such modifications to existing protocols could see an improvement in 3′ cDNA coverage [62]. While at the time of publishing there were no publicly available datasets or benchmarking of these developments, they look to improve full length cDNA recovery and throughput.

### Environmental metagenomics and viromics

Environmental genomics approaches like metagenomics and viromics are rapidly expanding our knowledge of viral diversity through the sampling of viral communities without cultivation. The requirement for clonal DNA has largely limited the application of first generation sequencing to viral isolates, or specific amplification of a low-titre target sequence from complex samples. While the seminal 2004 metagenomics study by Tyson et al. [63] was performed using random shotgun sequencing by capillary electrophoresis, its success was contingent upon genomes sampled from a low complexity environment. NGS approaches have enabled unbiased genome sequencing from high-diversity uncultivated environmental and clinical samples, following enrichment of virions (viromics) or depletion of host nucleic acids [64]. Viral and bacterial populations detected in metagenomics studies were initially analysed at the scale of the community; short fragment lengths generated by NGS posed challenges in identifying and assembling discrete viral genomes [65]. Although increases in NGS read length, and advances in computational methods have facilitated viral genome reconstruction from metagenomics data, assembly errors persist for highly fragmented, often non-overlapping and unevenly sampled sequences, particularly for low complexity regions [66–68]. The use of third generation sequencing in metagenomics studies can bypass assembly issues altogether, and as single DNA molecules are resolved, whole-genome phasing of polymorphisms is possible to glean information about viral diversity within a population. Early studies adopting long-read sequencing often employed hybrid approaches, combining third-generation platforms for assembly with NGS to enhance assembly accuracy [69,70]. Initially, nanopore metagenomics were used to rapidly characterise viruses with minimal library preparation, whereas SMRT-seq, although more expensive and lower throughput, was able to more accurately recover viral sequences using HiFi reads. As they mature, third-generation sequencing methodologies are constantly improving. Updated pores and chemistry supporting duplex reads on the ONT platform have seen marked improvements in accuracy, while recent changes to the library preparation for SMRT-seq offer improved throughput and affordability. Such advances would obviate the need for supplementation with NGS. With growing interest and evident benefits of third-generation sequencing approaches, benchmarking studies and datasets are emerging to inform their uptake in future metagenomics studies [70–72].

### Viral phylogenetics and taxonomy

Phylogenetic analysis is utilised in various applications across virology, including epidemiology, diagnostics, and taxonomy. The rapid accumulation of viral genome sequences, particularly from viromics and metagenomic approaches, has significantly influenced how viruses are classified. Traditionally, viruses were grouped based on phenotypic traits like their mode of replication, as in the non-hierarchical Baltimore system [73]. However, comparative genomics studies have since their inception, revealed unexpected evolutionary relationships among distant viruses, calling for a revision of classification based predominantly on phenotypic traits [74,75]. A significant challenge in viral phylogenetics is the rapid mutation rate of viral genomes, attributed to factors such as the low fidelity of viral replication machinery, recurrent homologous recombination events, and frequent horizontal gene transfer. Furthermore, when compared with cellular organisms, viral phylogenetics approaches need to account for vastly different evolutionary histories and polyphyly of viruses [76,77]. Recent revisions by the International Committee on Taxonomy of Viruses have expanded the hierarchical ranks from 5 to 15 and included metagenome-assembled genomes in official classification schemes [78,79]. These revisions aim to enhance the description of virus diversity and improve the alignment of viral taxonomy with host systems. With the widespread use of metagenomics and high-throughput genome sequencing approaches, coupled with advancements in computational methods for quantifying phylogenetic relationships, there is growing optimism for achieving a comprehensive and stable hierarchical taxonomy of viruses [80].

### Insertion site mapping of integrated proviruses

**Figure 3.**
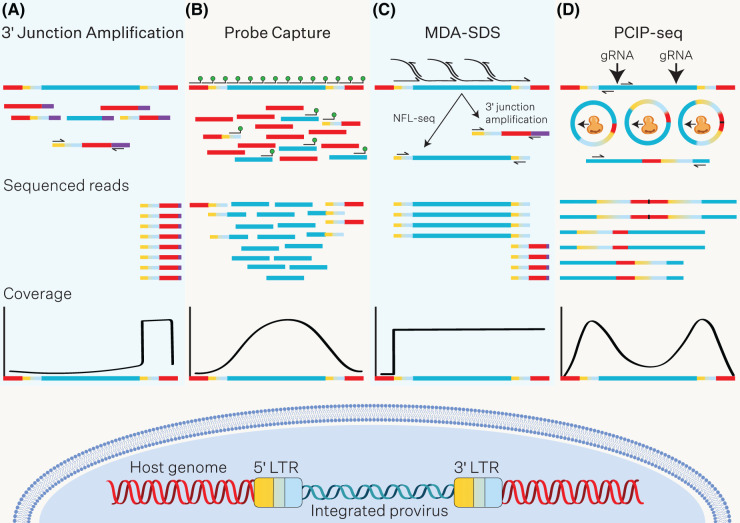
High throughput approaches to map proviral integration sites in the host genome. (**A**) 3′ junction amplification approaches were first used to map the junction of proviral integration sites of retroviruses like HTLV-1 and HIV in the human genome. DNA extracted from infected cells is subject to fragmentation by restriction enzymes or sonication, and then ligation to DNA linkers (purple). Integration sites can be amplified using one primer that binds to the 3′ viral LTR promoter and another that binds the linker. PCR products can then be prepared for sequencing by capillary electrophoresis or NGS. (**B**) DNA probe capture can enrich proviral integrants for NGS. A set of biotinylated DNA probes (green circle) is designed to tile the proviral genome. Probes that bind to the 5′ or 3′ end of the proviral genome, will often enrich for the junction of the integration site within the host genome. Infected genomic DNA is prepared for NGS using standard library preparation procedures. The libraries are then mixed with proviral-specific biotinylated probes for hybridisation. Streptavidin-coated magnetic beads are used to isolate the proviral DNA fragments and integration site junctions, which can then be subject to NGS. (**C**) Multiple-displacement amplification single genome sequencing (MDA-SGS) allows resolution of near full-length proviral sequences as well as mapping the integration site junction. DNA extracted from infected cells is diluted to a proviral endpoint, so that individual proviruses and integration sites can be independently amplified. MDA is catalysed by phi29 DNA polymerase, and from the MDA reaction, near full length (NFL) proviral genomes can be amplified by nested PCR and subject to capillary electrophoresis or long-read sequencing. Insertion sites can be amplified by 3′ junction amplification, and sequenced by NGS. (**D**) PCIP-seq leverages selective cleavage of circularised DNA fragments carrying proviral DNA with a pool of CRISPR guide RNAs, followed by inverse long-range PCR and long-read sequencing. Genomic DNA isolated from infected cells is sheared to approximately the length of the proviral genome. Intramolecular ligation is performed to create circular DNA, and remaining linear DNA is digested by nucleases. The circular DNA containing proviral sequences is selectively linearised by targeting regions adjacent to the 5′ and 3′ LTRs (black arrows) for CRISPR-mediated cleavage (orange). Inverse long-range PCR is performed to amplify the proviral integration site and proviral genome, followed by long-read sequencing.

Group VI retroviruses are single-stranded positive RNA viruses that are reverse transcribed into a DNA intermediate, which is chromatinised and stably integrated into the host genome. From there, it is transcribed, and transcripts are either packaged into new infectious virions, or translated into viral effector proteins. Extensive efforts have been made to characterise the retroviral insertion landscape and understand the functional implications of integration on both host and viral genomes [81–84]. Mapping the integration landscape in reservoirs of latent cells from chronic retroviral infections, such as HIV and HTLV-1, holds promise for monitoring latent clones in patients and identifying genomic contexts favourable for latency [83,85]. Retroviral insertion sites have typically been mapped by PCR based approaches to selectively amplify fragments containing viral and host junctions, followed by chain termination or high-throughput sequencing (Figure 3A) [84,86–88]. While such approaches have enhanced our understanding of retroviral integration, selection over time, and associations with latency, such enrichment methods are ripe for the introduction of biases and artefacts, and do not sequence the proviral genome [89]. This knowledge is critical in determining whether latent reservoirs of provirus are replication competent. Probe based proviral enrichment methodologies improve on sensitivity and specificity when compared with PCR based methodologies (Figure 3B) [90–92]. However, they do not yield contiguous, full-length proviral structural and sequence information. An elegant multiple-displacement amplification single genome sequencing (MDA-SGS) approach was developed to isolate and determine integration sites and full-length sequences of individual proviruses from genetically diverse clinical samples [93]. MDA-SGS is based on the principle of limiting endpoint dilution of gDNA, in which a single proviral integrant is assumed isolated. The diluted genetic material is amplified by phi29-catalysed multiple displacement amplification, followed by selective amplification of the near full-length provirus and sequencing by SMRT-seq, in parallel to insertion site mapping using PCR based approaches (Figure 3C). More recently a parallel RNA, integration, and proviral (PRIP) sequencing protocol expanded on MDA-SGS to include measurement of proviral expression by digital droplet PCR [82]. PRIP-seq is founded on a limiting dilution of infected cells to isolate individual proviral integrants, and similarly uses MDA prior to amplification of the proviral genome, alongside simultaneous integration site loop amplification, the products of which are subject to NGS.

Long-read whole genome sequencing of infected host DNA can provide comprehensive mapping of native proviral integrants, spanning both integration junctions and resolve hallmarks of retroviral integration such as target site duplication, along with epigenetic information [94]. Such unbiased whole genome sequencing approaches have been applied to bacterial genomes to map prophage integration, and have been shown to be useful in tracking outbreaks in food processing environments, understanding host-viral interactions and micro-evolution events within a population, and resolving closely related bacterial strains within a sample [95–97]. However, in eukaryotic hosts, whole genome sequencing approaches to map proviral integrants require deep sequencing to map sufficient insertion events, which can be prohibitively expensive, particularly in a clinical setting. Thus, there is keen interest in developing enrichment strategies that can capture both the proviral genome and integration sites for long-read sequencing from heterogeneous samples. To this end, a pooled CRISPR inverse PCR-sequencing (PCIP-seq) protocol has been developed, which enables enrichment of proviral integrants for nanopore sequencing [98]. PCIP-seq utilises CRISPR-Cas9 mediated cleavage of circularised DNA fragments containing proviral integrants, followed by inverse long-range PCR and multiplexed sequencing by nanopore (Figure 3D). While this method has proved effective in recovering insertion sites for pathogenic retroviruses including HIV and HTLV-1, the recovery of full-length proviral genomes was low, and because the approach employs PCR amplification, DNA modifications are lost. Many elegant enrichment protocols using CRISPR-Cas9 are being developed for use with long-read sequencing, which in the future could be modified to enrich for proviral integrants in their native genetic and epigenetic context [99–102].

### Genomic epidemiology

Viral genome sequencing has transformed our ability to monitor viruses crucial to the health of ecosystems, agriculture, and human populations. While known viruses are monitored for outbreaks, the potential threat posed by the zoonotic transmission of unknown viruses remains a significant concern [103,104]. Co-ordinated efforts have been made to collect and analyse viral genomes, to predict and prepare for potential outbreaks [105–107]. Comparison between whole viral genomes enables mapping of genetic complexity to monitor epidemics, identify chains of transmission, and to detect variants associated with increased virulence or immune evasion. There is a rich history of genomic epidemiology using both targeted and unbiased sequencing approaches (reviewed in [108,109]). Here we will discuss the collective viral sequencing efforts that enabled a rapid and effective response to the SARS-CoV-2 pandemic, culminating in the largest repository of viral genomes to date.

Although coronavirus disease (COVID-19) was first detected in humans in 2019, its emergence had long been predicted. SARS-Coronavirus (CoV) and MERS-CoV broke out in human populations in the early 21st century, spilling over from bats and camels (via bats), respectively [110]. These outbreaks underscored coronaviruses as potential pandemic pathogens and emphasised the importance of understanding coronavirus reservoirs. Noting the paucity in coronavirus sequences from bats, a 2017 study geared at identifying emerging pandemic threats sampled almost 20 000 bats, rodents and humans, amplified coronavirus genes, and subjected them to chain termination sequencing for comparative analysis [111]. The authors specifically noted that this approach was adopted to facilitate viral discovery in resource-poor settings, which are often predicted hotspots of disease emergence, and where high throughput sequencing infrastructure is largely unavailable. Scientific resources and infrastructure disparities should be a primary consideration in pandemic preparedness responses, to ensure practices are accessible for effective implementation. Within weeks of the first reported COVID-19 cases in humans, a full-length sequence of the SARS-CoV-2 genome had been generated by total RNA sequencing on the Illumina platform using a clinical metagenomics approach, allowing assembly and analysis of the contigs to identify a potential aetiological agent [112]. This approach produced the 29 875 bp genome sequence, which thanks to years of preparedness efforts, could be identified as closely related to BatCoV RaTG13, found in bats. The sequence was deposited on Genbank on the 10th January 2020. Within hours, the sequence was being used to develop vaccines, which would markedly help global public health efforts to control the virus.

Resolution of the SARS-CoV-2 genome allowed for the design of RT-PCR diagnostic assays, which were implemented throughout the COVID-19 pandemic. Given the rapid global spread of SARS-CoV-2 and the profound public health, social, and economic impacts of COVID-19, there was a need for whole genome sequencing of SARS-CoV-2 alongside routine diagnostics [113]. Sequence information allowed the phylogenetics of outbreaks to be closely monitored to identify transmission networks and infer the origin of many cases [114]. Tracking viral evolution over time informed public health responses, and continues to have implications for understanding immunity and vaccine development [115–118]. The global reach of the SARS-CoV-2 pandemic saw many countries that had not previously adopted genomic surveillance methodologies generating and using genomics data. With over 16 million SARS-CoV-2 genomes deposited on GISAID in 2024, we have seen almost every conventional sequencing approach applied to SARS-CoV-2. Of note was the widespread adoption of ONT to sequence SARS-CoV-2 [119,120]. The nanopore platform offers many features that make it suitable in a pandemic response; namely, the minimal investment capital and laboratory infrastructure required to implement ONT devices, real-time data generation and analysis, the relative ease of library preparation, and the portability of ONT devices. While setup costs are significantly lower than other approaches, the running costs are on par with Illumina, which is an important consideration in low resource environments. Nonetheless, these features saw the successful implementation of nanopore sequencing in the field in previous efforts to monitor the Ebola virus epidemic in West Africa in 2015, and the Zika virus epidemic in the Americas in 2016 [121–123]. For genomic epidemiology, base-level resolution is critical for understanding viral evolution and monitoring chains of transmission; erroneous annotation of mutations arising due to sequencing errors would significantly confound the interpretation of viral phylogenies. Given a limitation of nanopore sequencing is base-resolution accuracy, this was of significant concern. To this end, a benchmarking study was published early in the pandemic to define requirements for accurately monitoring SARS-CoV-2 outbreaks with ONT [124]. They showed that nanopore sequencing allowed highly accurate detection of SNVs in patient isolates from consensus sequences with a 60× sequencing depth. While nanopore is suitable for the detection of large structural variations, error rates preclude accurate detection of small indel variants and rare SNVs. These findings demonstrated the suitability of ONT for routine phylogenetic analysis in viral surveillance. The reduced sensitivity for rare variants and small structural variations supports the use of NGS methods for studies of virus evolution and pathogenicity. This benchmarking study importantly removed barriers for adoption of ONT in viral epidemiology. In subsequent years significant improvements in ONT accuracy of low complexity regions may have addressed many of the observed shortcomings, and their benchmarking will be of importance for future genomic epidemiological efforts.

### Viral sequencing in the clinic

Genome sequencing of human pathogenic viruses like HIV, influenza, and hepatitis B and C serves not only research purposes but also crucial clinical applications, particularly in accurate and rapid diagnostics when serological results are uncertain [125–127]. Clinical sequencing of hepatitis B virus has been used to inform and monitor treatment responses over time, and to distinguish between acute and chronic infection [128–130]. However, the primary clinical application of viral sequencing lies in the detection and monitoring of drug resistance, best exemplified by RNA viruses like HIV. HIV is managed by combination antiretroviral therapy, with agents that target the reverse transcriptase, integrase or protease components of the HIV replication pathway. Despite treatment, low-level viral replication can persist, leading to the emergence of drug-resistance [131]. RNA dependent polymerases such as reverse transcriptase are highly error prone, giving rise to diverse viral quasispecies within an individual patient [132]. Sequencing of the gene encoding the enzymatic HIV pol has been adopted for detecting resistant quasispecies, and allows an informed approach to altering treatment courses to improve disease outcomes [133].

While sequencing subgenomic fragments or single genes can detect dominant resistant species, minor variants with low allelic frequencies often remain undetected, as chain termination methods typically have a detection limit of ∼30% of the population [134]. These low-frequency variants can hold significant clinical implications for viral resistance and other phenotypic traits like receptor tropism. High-throughput sequencing can detect low allele frequency quasispecies, and although the cutoff recommended by the WHO guidelines is a conservative 20%, studies have shown that deep sequencing can detect quasi-species frequencies of down to ∼1% [134]. However, despite the accuracy and sensitivity offered by NGS, assembling full-length HIV genomes remains challenging. Long-read approaches offer a more tangible means to resolve haplotype genomes of HIV quasispecies, enabling linkage analysis of multiple resistance-conferring mutations, to better understand the mutational landscape and resistance mechanisms [135–137]. With ONT duplex sequencing and SMRT HiFi sequencing available, accuracy rates are nearing NGS accuracy. To sequence the viral quasispecies with sufficient depth, most high-throughput methodologies still employ an amplification or enrichment step, which imposes biases, and primer site degradation is a concern, particularly with RNA viruses like HIV. Adaptive sampling on the ONT platform is a real-time software controlled method to preferentially sample target sequences, and could be employed to enrich viral genomes. Currently adaptive sequencing is not efficient with low-copy number sequences, such as in HIV infection, however when paired with an amplicon-seq approach, could be powerful [138]. While clinical metagenomics approaches could offer unbiased profiling of the mutational landscape, the depth of sequencing required to sample even high-abundance quasispecies in low-proviral load infection is untenable. Moreover, implementation of unbiased sequencing also raises ethical concerns regarding incidental findings, which is an important conversation occurring in medical genomics communities more broadly [139]. Currently, whole genome sequencing for HIV genotyping is primarily applied in research settings, due to cost considerations when compared with gene-centric chain-termination approaches, as well as non-standardised and often laborious computational pipelines for mapping, detection, assembly and analysis of quasispecies. As it stands, the potential for its uptake in the clinic is attractive to enable earlier detections of therapeutically relevant quasispecies in infectious disease. However, new and standardised approaches are required to resolve issues of cost, sensitivity, and analysis, to enable wide-spread, regulated clinical application of viral whole genome sequencing.

## Perspectives

- Viral sequencing is a critical methodology enhancing understanding of molecular virology, and also in molecular epidemiology and clinical virology. The significance of viral sequencing was underscored during the recent SARS-CoV-2 pandemic, where it was instrumental in diagnostics, infection control, and therapeutic development.
- The practice of viral genomics dates back to the inception of genome sequencing itself. Various methods exist for sequencing viruses, each tailored to the specific virus under investigation and its intended application. Third-generation sequencing platforms are now being increasingly employed to produce single-molecule, contiguous, and haplotype-resolved viral genomes in research settings. Initial concerns regarding accuracy and throughput are being addressed, with benchmarking studies defining the optimal conditions for generating and analysing meaningful long-read data. These advancements pave the way for the widespread adoption of long-read sequencing in applied settings, including clinical practice.
- Third-generation sequencing technologies offer unprecedented insights into the molecular diversity within viral populations. With the integration of functional genomics assays into long-read platforms, we anticipate studies delving into the functional consequences of this molecular diversity. In clinical settings, the potential for personalised medicine to tailor treatment regimens based on viral mutational profiles holds promise for improving patient outcomes. However, to fully realise this potential, standardisation of data processing and interpretation will be paramount, alongside experimental models for validation.

## Open Access

Open access for this article was enabled by the participation of University of Queensland in an all-inclusive *Read & Publish* agreement with Portland Press and the Biochemical Society under a transformative agreement with CAUL.

## Abbreviations

DRS: direct RNA sequencing
HPV: human papillomavirus
MDA-SGS: multiple-displacement amplification single genome sequencing
NGS: next-generation sequencing
ONT: Oxford Nanopore Technologies
PCIP-seq: pooled CRISPR inverse PCR-sequencing
PRIP: parallel RNA, integration, and proviral
SMRT: single-molecule real-time
ZMW: zero-mode waveguide
